# Dry Cold Forging of Pure Titanium Wire to Thin Plate with Use of β-SiC Coating Dies

**DOI:** 10.3390/ma13173780

**Published:** 2020-08-27

**Authors:** Tatsuhiko Aizawa, Tomoaki Yoshino, Tatsuya Fukuda, Tomomi Shiratori

**Affiliations:** 1 Surface Engineering Design Laboratory, Shibaura Institute of Technology, Tokyo 144-0045, Japan; 2 Komatsu-Seiki Kosakusho, Co., Ltd., Nagano 392-0022, Japan; yoshino@komatsuseiki.co.jp; 3 Tokai Engineering Service, Co., Ltd., Gifu 504-0852, Japan; t-fukuda@tes2001.com; 4 Faculty of Engineering, University of Toyama, Toyama 939-8202, Japan; shira@eng.u-toyama.ac.jp

**Keywords:** SiC-coated SiC die, dry cold forging, titanium, high reduction rate, surface area extension rate, contact interface, titanium oxide debris film, carbon isolation

## Abstract

Dense β-SiC coating with 3C-structure was utilized as a dry cold forging punch and core-die. Pure titanium T328H wires of industrial grade II were employed as a work material. No adhesion or galling of metallic titanium was detected on the contact interface between this β-SiC die and titanium work, even after this continuous forging process, up to a reduction in thickness by 70%. SEM (Scanning Electron Microscopy) and EDX (Electron Dispersive X-ray spectroscopy) were utilized to analyze this contact interface. A very thin titanium oxide layer was in situ formed in the radial direction from the center of the contact interface. Isolated carbon from β-SiC agglomerated and distributed in dots at the center of the initial contact interface. Raman spectroscopy was utilized, yielding the discovery that this carbon is unbound as a free carbon or not bound in SiC or TiC and that intermediate titanium oxides are formed with TiO_2_ as a tribofilm.

## 1. Introduction

Pure titanium has been utilized for metallic parts and tool components in various medical and biomedical applications due to its well-defined bio-compatibility [1]. However, its metal forming and forging often suffers from high friction and wear by its severe adhesion or galling [2]. Since heat is generated—even during cold forging—at the contact interface between die and work materials, this galling behavior is enhanced when the interface temperature exceeds the critical temperature [3]. Hence, the reduction in thickness in its forming and forging must be lowered as much as possible to be free from galling, when using the tool steel dies or tungsten carbide cobalt tools [4]. As pointed out in [5,6,7], this titanium galling process advances in two mechanisms. The fresh titanium surface adheres to the die surface by mass transfer so that the friction coefficient abruptly increases and results in seizure. The titanium oxide debris particles splash from the work material surface to the air, deposit on the die surface and become a hard lock to hinder the further forming, or to damage the die surfaces. These studies suggest that other die materials, rather than tool steels, tungsten carbide cobaltWC(Co) and sintered ceramics must be selected for metal forming and forging of titanium works, otherwise, the pure titanium is difficult or nearly impossible to be near-net shaped into a product due to this severe galling.

Thick SiC (Silicon Carbide) coating was selected as a die material for upsetting the pure titanium wires and bars [8,9]. After microstructural analysis, no adhesion of metallic titanium was detected on the SiC coating punch and die surfaces, even after continuous upsetting by fifteen shots. A SiC-coated SiC die with a triangular cavity was utilized to describe the dry cold forging behavior, increasing the reduction in wire diameter by up to 35% [10,11]. A titanium wire was successfully forged in cold and dry conditions to a triangular one without galling. Titanium oxide debris deposits on the SiC coating punch and form a thin surface layer, enough to continue the forging process. After [11,12] this galling-free forging process was sustained by the carbon isolation from β-SiC coating. The carbon agglomerates were detected only at the center of the initial contact surface between the SiC coating and titanium work. This suggests that selection of β-SiC coating as a die is a suitable solution to dry galling-free cold forging with a higher reduction in thickness than 50%.

In the present paper, these β-SiC-coated SiC punch and core-die are prepared for dry cold forging of pure titanium T328H wires of industrial grade II, up to a reduction in diameter by 70%. This high reduction forging becomes an essential step to shape the blank plates for glass-frames from titanium wires and bars in a single shot [7]. In the cold forging experiments, a CNC (computer numerical control) stamping system is employed to upset the pure titanium wire by controlling the applied stroke in each reduction. The power-to-stroke relationship is in situ monitored to describe this dry cold upsetting process. The surface area extension rate monotonously increases by the flattening of a circular wire to a thin plate without significant bulging deformation. The applied power also increases monotonically with the work hardening of titanium. SEM (Scanning Electron Microscopy), EDX (Electron Dispersive X-ray spectroscopy) and Raman spectroscopy were utilized to describe the contact interface condition between SiC coating and titanium work. After continuously forging in cold and dry conditions up to a 70% in reduction, a tribofilm is in situ formed radially from the center line of this contact interface. The carbon agglomerates isolated from β-SiC coating distribute in dots only at the center of contact interface. Intermediate titanium oxide thin films are formed in this tribofilm together with TiO_2_ debris. These two mechanisms are responsible for the galling-free cold forging of titanium and titanium alloys with a high reduction and lower friction.

## 2. Experimental Procedure

The SiC-coated SiC punch and core-die was prepared for dry cold forging experiments with a high reduction in thickness. These punch and die were fixed into the upper and lower die sets. A CNC stamping system was utilized for this experiment with the specified loading sequence. During loading, the power to stroke relationship was in situ monitored to describe the dry forging behavior, up to the reduction in wire diameter by 70%. 

### 2.1. SiC-Coated SiC Dies

**Figure 1:**
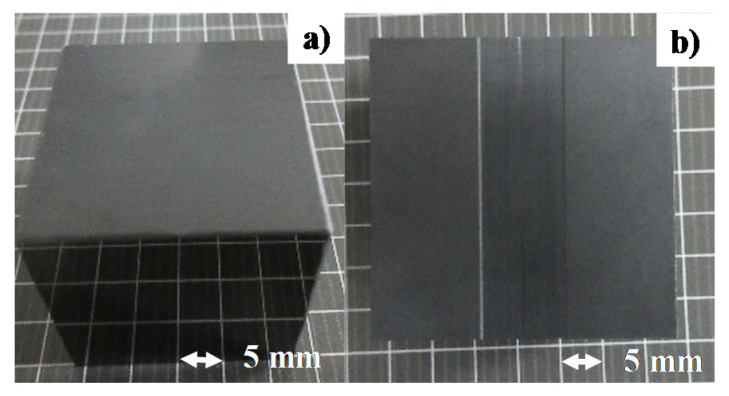
SiC-coated SiC punch and die. (**a**) SiC-coated SiC punch, and (**b**) SiC-coated SiC die with a rectangular cavity.

Thick β-SiC coating was deposited onto the sintered SiC substrate by thermal CVD (chemical vapor deposition) process at 1500 K. The coating thickness was 4 mm. Both the punch head and core die were polished and cleansed by the ultrasonic cleaner (200DL, Kaijyo, Tokyo, Japan). This SiC-coated SiC substrate was directly utilized as a punch, as shown in Figure 1a. The diamond sawing was used to cut the rectangular groove as a cavity of core-die, as depicted in Figure 1b. In the following experiment, the pure titanium wire is forged into this cavity with the depth of 0.15 mm and the width of 10.0 mm. A half circular groove with the diameter of 0.3 mm was also cut into the bottom of groove to fix the circular titanium wire work. No cracks or defects were present, even after thermal history in CVD and mechanical finishing.

### 2.2. Dry Cold Forging Process

**Figure 2:**
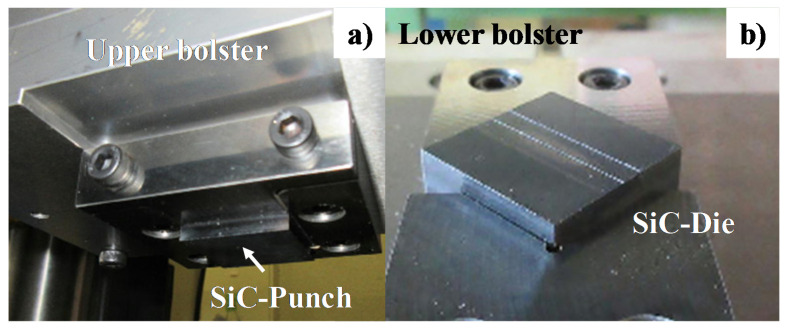
Upper and lower cassette die system. (**a**) Upper die set with SiC-coated SiC punch, and (**b**) lower die set with SiC-coated SiC die.

**Figure 3:**
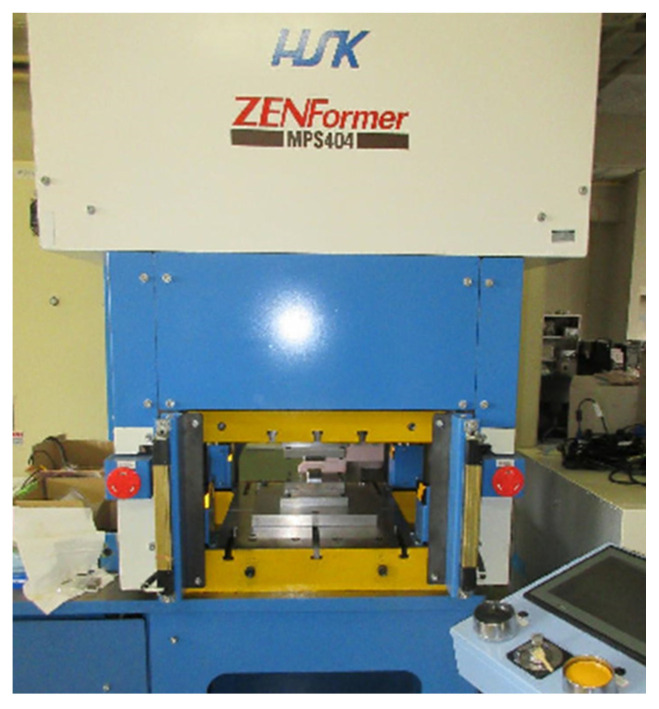
Computer numerical control (CNC) stamping system for dry cold forging of the pure titanium wire.

These SiC-coated SiC punch and core die were fixed into the upper and lower die sets, respectively, as shown in Figure 2. This die set was cemented to upper and lower bolsters of a CNC stamping system (MPS404 Zen-Former, HSK, Yokohama, Japan). Figure 3 depicts the CNC stamper with four motor-driving units, where each unit works independently to adjust the eccentricity in loading. Among several candidate programs, the stroke was lowered in a constant velocity of 0.1 mm/s down to the specified minimum stroke (δ_m_), held for 1 s and then moved up to the original position at the same velocity. This δ_m_ was varied for each reduction in wire diameter. No interface delamination occurred before or after cold forging. To be discussed later, the estimated maximum forging stress in local is 10 GPa, lower than the compressive strength of 30 GPa for SiC coating.

A pure titanium T328H wire in the industrial grade II was employed as a work material in the following forging experiments. Its diameter was 0.98 mm and its length was 10 mm. The chemical composition of this T328H wire consists of hydrogen by 0.0012 mass %, oxygen by 0.097 mass %, nitrogen by 0.007 mass %, iron by 0.042 mass %, carbon by 0.007 mass % and titanium for balance.

### 2.3. Contact Interface Analysis

SEM–EDX (JOEL, Tokyo, Japan) and a three-dimensional profilometer were utilized to analyze the contact interface condition between the SiC coating and the titanium work. In particular, two regions were selected in the contact interface to describe the difference in the titanium oxide debris film formation and in the carbon isolation from the SiC coating. Raman spectroscopy (Nihon-Kogaku, Co., Ltd., Tokyo, Japan) was used to analyze the binding state of silicon, carbon, titanium and oxygen on the contact interface.

## 3. Experimental Results

Dry cold forging with high reduction in thickness is performed to investigate the material flow during loading. The power–stroke relationship is in situ monitored to describe the flattening process from a circular wire to a thin plate. The surface area extension rate is estimated and employed as a parameter to analyze the formation of the fresh surface in this flattening behavior. SEM–EDX analyses as well as Raman spectroscopic analyses were utilized to describe the titanium oxide debris film formation on the contact interface and to investigate the role of free carbon agglomerates from the SiC coating on the solid lubrication under dry cold forging with a high reduction.

### 3.1. Cold Forging of Titanium Wire to Plate

A circular pure titanium wire was forged in dry and cold conditions, up to each specified reduction in thickness. During this forging process, the wire was placed at the micro-groove in the cavity of SiC-coated SiC die.

**Figure 4:**
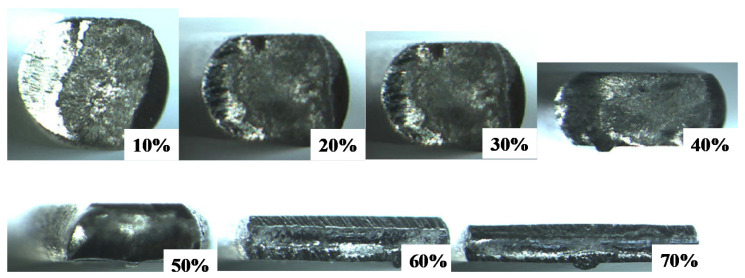
Variation of cross-sectional geometries from circular wire to flat plate with increasing the reduction in diameter.

**Figure 5:**
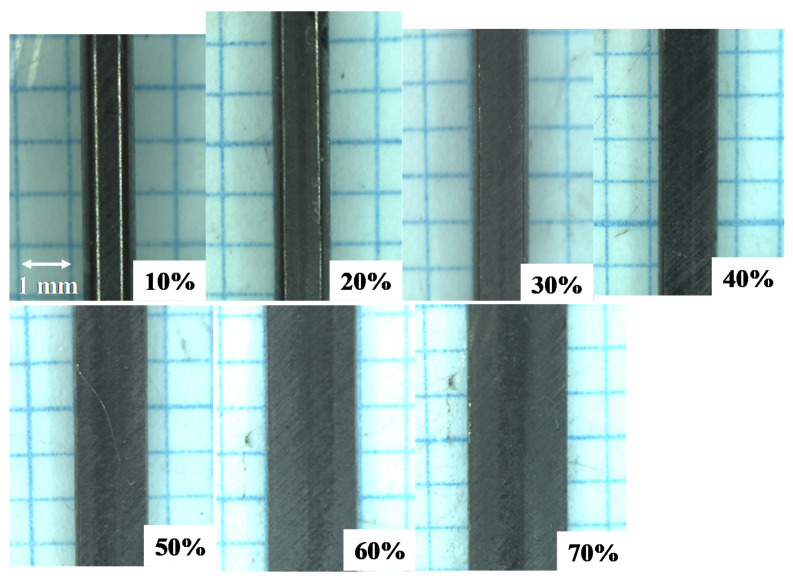
Variation of contact interface area in flattening into a plate with increasing the reduction in diameter.

Figure 4 depicts the variation of the titanium work cross-section from a circular wire with a diameter of 0.98 mm to a flat plate with a thickness of 0.3 mm and width of 2.3 mm by increasing the reduction in thickness (r) by up to 70%. A circular wire is first compressed to deform it elasto-plastically in the axial direction and is continuously upset during a reduction in thickness of more than 20%. This change in deformation mode from the axial compression to the flattening process is also understood by observation on the plain view of forged wire. Figure 5 shows the variation of plain view of forged titanium wires with increasing the reduction. Up to r = 10%, the axial compression takes place together with flattening along the contact interface. In reductions further than 10%, the flattening of wire mainly drives this cold forging. The reduction in wire thickness results in the broadening of wire in the lateral direction.

When using the SKD11 punch and core die, the bulging process dominates this upsetting behavior of the titanium wire because of significant friction on the contact interface. As seen in both Figure 4 and Figure 5, no significant bulging of wire was seen in its cross-section and plane view. This implies that monotonous flattening is driven by uniform plastic flow velocity of wire in the lateral direction and that the friction coefficient on the contact interface is greatly reduced when using the SiC-coated punch and core-die. After [13], the friction coefficient is estimated to be 0.1 to 0.2 from small bulging ratio in Figure 4 and Figure 5. When r > 20% in Figure 5, the fresh surfaces are detected as a metallic shining part at the center of forged specimen by optical microscopy observation.

The surface area extension ratio (e) is employed as a parameter to describe this flattening process under the assumption that the work material flow is in the plain strain condition. Then, e is defined by the ratio of the current side surface area (A) of forged wire to the original side surface area of circular wire (A_0_); e.g., e = A/A_0_. A is estimated for simplicity by A = 2 (W + H) × L for the current flattened work width (W) and height (H) during cold forging. A_0_ is defined by πd × L for the diameter (d) and length (L) of the wire before forging.

**Figure 6:**
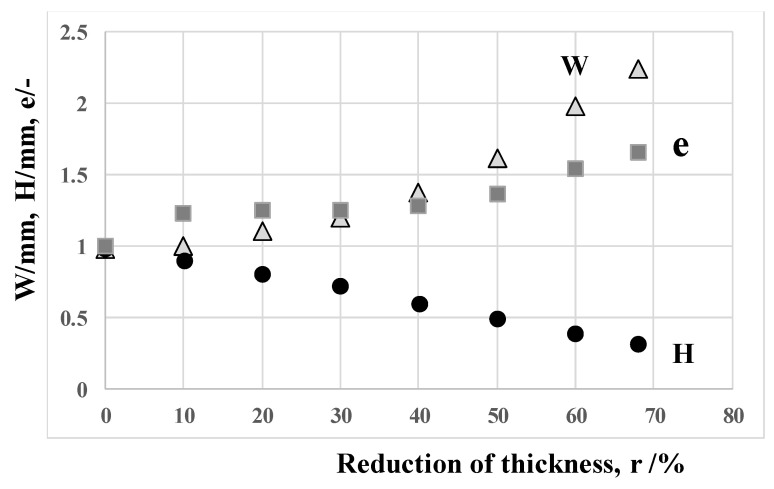
Variation of the titenium wire thickness (H), the width (W) and the surface aea extension rate (e) with increasing the reduction of thickness.

Figure 6 shows the variation of measured W and H as well as the estimated e with increasing the reduction in thickness (r). W and H monotonously increases and decreases, respectively, with r. The surface area extension ratio significantly increases when r > 30%. This is just corresponding to the appearance of the fresh titanium surface on the contact interface of forged work in Figure 5. That is, the fresh titanium surface from the inside of work is in contact with the SiC coating punch during flattening process in cold forging for r > 30%. The smooth surface of forged titanium work in Figure 4 and Figure 5 prove that no metallic transfer or galling takes place from this titanium fresh surface to the SiC coating punch head.

### 3.2. Power to Stroke Relationship

**Figure 7:**
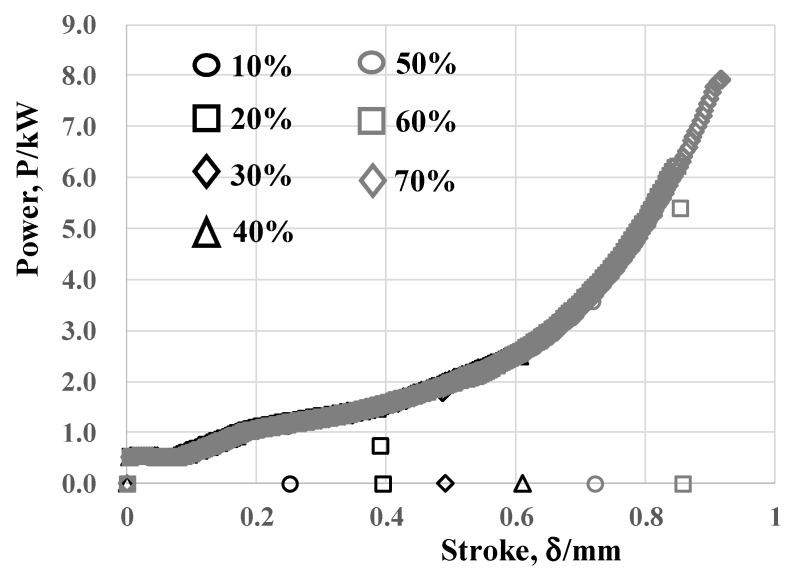
Power to stroke relationship in dry cold forging of pure titanium wire at the reduction in diameter by r = 10%, 20%, 50%, and 70%.

Variation of the applied power with increasing the stroke in cold forging was in situ measured up to each specified reduction in thickness by 10%, 20%, 30%, 40%, 50%, 60% and 70%. These relations are plotted into the power-to-stroke relationship in Figure 7. Each measured relation between the power (P) and the stroke (δ) for δ < δ_m_ superposes on each other to be edited into a single master curve. This single power–stroke relationship represents for the elasto-plastic flow intrinsic to pure titanium in the cold forging. In addition to the initial elasto-plastic deformation of wire in compression for r < 20%, this forging behavior is governed by the flattening flow of titanium along the SiC coating punch surface for r > 20%. In particular, the applied power increases exponentially with the stroke for r > 30% since the contact area increases between the forged titanium work and the SiC coating punch.

### 3.3. Contact Interface Analysis

**Figure 8:**
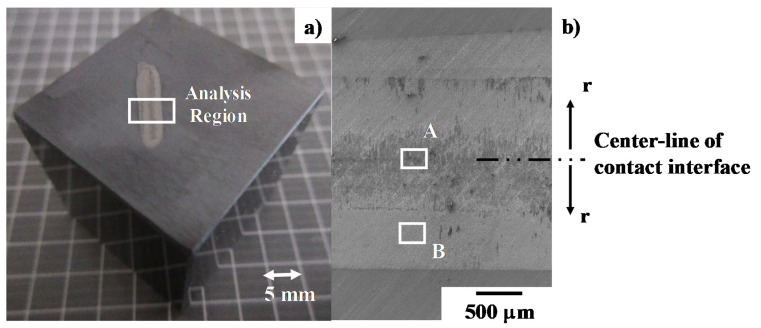
Optical microscopy and SEM analyses on the contact interface conditions of SiC-coated SiC punch after dry cold forging up to r = 70% for 10 shots. (**a**) Optical microscopy image on the SiC coating after cold forging test, and (**b**) SEM images on the black and white zones.

SEM–EDX was first employed to describe the contact interface conditions between the SiC coating and the titanium wire after continuously forging up to the reduction in thickness by 70% in twenty shots. Figure 8 depicts the optical microscopy and SEM images on the contact interface. As had been reported in [12,14], this contact interface consists of two regions in Figure 8a; e.g., a black zone at the center of the contact interface and white zones sandwitching this black zone.

Figure 8b shows the low resolution SEM image on this contact interface. These two zones are formed in the radial direction from the center line of the initial contact interface between the titanium wire and SiC coating. The black zone consists of black and dark gray layers growing in the radial direction. This black zone width is equivalent to the fresh surface width in Figure 5; the formation of this black zone has close relation to the flattening process in the cold dry forging. In addition, pure titanium work flow in Figure 4 and Figure 5 with less bulging behavior implies that this black zone could be a tribo-film to suppress the adhesion of fresh surface and to reduce the friction coefficient against the flattening plastic flow.

**Figure 9:**
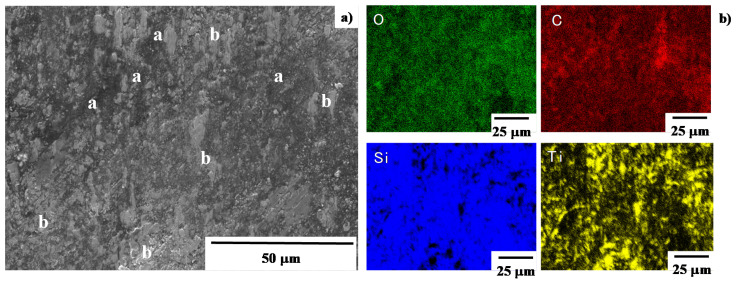
SEM–EDX analyses on the contact interface at the A-region. (**a**) SEM image on the A-region, and (**b**) element mapping of oxygen, carbon, silicon and titanium on the A-region.

SEM–EDX analyses were first utilized to describe the microstructure and element mapping of the A-region in Figure 8b. As shown in Figure 9a, the microstructure of black zone at the center of A-region consists of two zones; e.g., black a-zone and gray b-zone. Element mapping of the same analysis region as seen in Figure 9a, was depicted in Figure 9b. The a-zone in Figure 9a is an agglomerate with high carbon contents on the SiC coating surface. Since the titanium and oxygen maps are coincident with each other at b-zones, this b-zone is a titanium oxide layer also on the SiC coating. Since no overlapping was detected between carbon and titanium maps, these a and b-zones are exclusively formed on the contact interface of the SiC coating punch surface. The black zone consists of the titanium oxide films and the carbon agglomerates.

**Figure 10:**
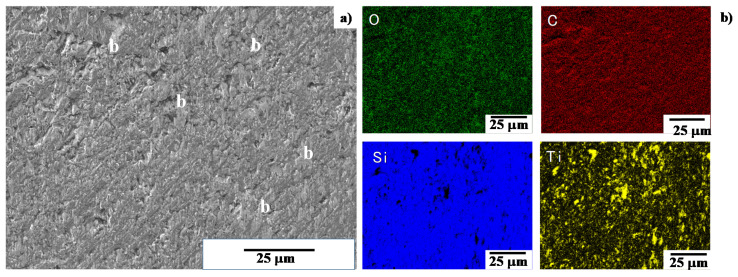
SEM–EDX analyses on the contact interface at the B-region. (**a**) SEM image of the B-region, (**b**) element mapping of oxygen, carbon, silicon and titanium on the A-region.

SEM image and element mapping of the B-region in Figure 8b, are shown in Figure 10. Uniform carbon mapping reveals that no carbon agglomerates are formed on this B-region, far from the center of contact interface. Since the titanium map is also coincident with the oxygen one, titanium oxide films are sparsely formed on the SiC coating as a b-zone in Figure 10a. This SEM–EDX analysis proves that the black and gray zones in Figure 8b are carbon agglomerates and titanium oxide films, respectively. They are formed as a thin film on the interface between the SiC coating and pure titanium work.

### 3.4. Raman Spectroscopy Analysis

**Figure 11:**
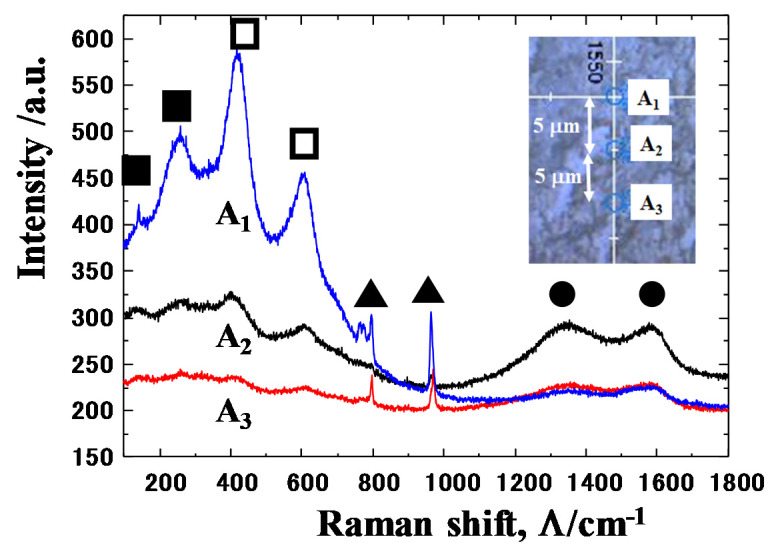
Raman spectrum of the carbon agglomerates, SiC and titanium oxide on the A-region of contact interface between β-SiC coating and titanium work.

Raman spectroscopy was employed to locally characterize the carbon agglomerates as well as the titanium oxide thin films on the contact interface. Figure 11 shows the Raman spectra measured by wide scanning at the A-region of contact interface between β-SiC coating and titanium work. Three positions (A_1_, A_2_, and A_3_) were selected for Raman spectroscopy analysis; e.g., A_1_ was located in the b-zone in Figure 9a, A_2_, in the a-zone and A_3_, near the b-zone. The distance among A_1_–A_3_ was constant by 5 βm. As in the literature [15,16,17,18], and the previous study in [14], two broad D and G-peaks are detected at the Raman shift of Λ = 1300 cm^−1^ and 1600 cm^−1^, respectively. This proves that a-zones in Figure 9 are not titanium or silicon carbides but they are agglomerates of free or unbound carbon. That is, free carbon is present at the center of the contact interface in correspondence to high carbon agglomerates in Figure 9b.

Two narrow peaks at Λ = 800 cm^−1^ and 950 cm^−1^, respectively, with weak intensities reveal that β-SiC punch surface coexists with a tribofilm. Compared to high intensity peaks at β = 800 cm^−1^ and 950 cm^−1^ just outside of the contact interface, their peak intensities are much smaller. This implies that the contact interface on the SiC coating punch is mainly covered by the tribofilm as well as the carbon dots. Other peaks than these two include a broad peak detected around Λ = 200~600 cm^−1^. If TiO_2_ were present as a titanium oxide in the interface, the narrow peaks could be detected at Λ = 400 cm^−1^ and 600 cm^−2^, respectively. No detection of narrow peaks implies that titanium oxide film consists of various binding states in the titanium - oxygen system from TiO to TiO_2_ through the intermediate Magneli phase oxides. In particular, other broad peaks at Λ = 150 and 250 cm^−1^ suggest that the intermediate titanium oxides are formed together with TiO_2_ debris particles in the tribofilm.

The Raman spectrum at A_1_ reveals that b-zone on the contact interface mainly consists of a titanium oxide film on the SiC coating with less free carbons. The Raman spectra at A_2_ and A_3_ show that the a-zone mainly consists of the free carbon dots with less formation of titanium oxide film. This Raman spectroscopy proves that free carbon agglomerates are formed on the contact interface away from the titanium oxide tribo-film..

Raman spectroscopy as well as SEM–EDX analyses prove that the free carbon agglomerates with the size of 10 m work as a solid lubricant to prevent the SiC coating punch from adhesion to the titanium fresh surface and to lower the friction on the interface even at its center. The Magneli-phase intermediate titanium oxide thin tribofilms also play as a lubricious layer on the contact interface.

## 4. Discussion

Dry cold forging with a higher reduction in thickness than 50% is necessary for near-net shaping of titanium wires or bars for medical parts or tools. This shaping is also accompanied with a higher surface extension area ratio than 1.5. This implies that a fresh surface comes out of the inside of the titanium work and contacts with the die surfaces with the risk of its adhesion or galling to tools and dies. The present dry cold upsetting with the use of SiC-coated SiC punch and core-die proves that cold dry forging with a reduction higher than 50% is put into practice with low friction and without galling. This significant difference in cold forging performance from the conventional process with the use of tool steel and WC (Co) dies is attributed to the formation of titanium oxide tribofilm as well as the solid lubrication by the free carbon agglomerates.

In the conventional forging, hard TiO_2_ debris particles splash from the deforming work surface, deposit onto the die surfaces and lock the flattening process in forging [7]. The titanium fresh surface directly adheres onto the die surfaces to stop the forging process at the lower reduction level. The tribofilm formed on the contact interface consists of the Magneli-phase intermediate titanium oxides, as well as TiO_2_. As reported in [19,20], these intermediate oxides TiOx for 1 < x < 2 elasto-plastically deform as a lubricious oxide to form a thin tribofilm. The thin tribofilm grows radially from the center line of the contact interface in Figure 4, Figure 5 and Figure 8a; this trace proves this lubricious elasto-plastic flow of intermediate titanium oxide films.

As had been studied in [11,12,14], no carbon sources are present in titanium work. In addition, the bound carbon in the β-SiC crystals, is difficult to unbind even at high stress transients in cold forging [21]. On the other hand, the carbon-supersaturation into SiC layers drives their growth during the high temperature process as suggested by [22]. A supersaturated carbon solute is easy to isolate from the carbon-supersaturated SiC and to form its agglomerated dot by its grain boundary diffusion. As shown in Figure 9 and Figure 11, no free carbons are present on the SiC coating surface except for the center line of the contact interface, which is subjected to high stress transients in forging. This suggests that free carbon solutes are forced to diffuse by this stress gradient through the grain boundaries to the contact interface even in atmospheric and room temperature conditions. Assuming that a titanium wire is elastically in contact with the SiC coating, with an initial contact area of 100 μm × 10 mm accompanied by a load of 10 kN, the applied normal stress on the contact surface reached to 10 GPa. The titanium work deforms elasto-plastically without galling in the presence of the free carbon solutes, isolated from highly stressed SiC crystals by diffusion under this high stress gradient.

The reduction in thickness in cold forging with the use of conventional dies is strictly limited to be less than 30% so that hot incremental forging processes with thermal annealing are necessary to shape the circular wire and bar to thin plates for further near-net shaping. When using the SiC coating punch and core-die, the wire can be shaped into a flat plat by a single-shot cold forging. This saving of processing and production time is attractive to practical operations in forging. In addition, the forged titanium plate has fresh and smooth surfaces ready to be directly shaped into the products by the successive steps in fine forging. SiC-coated tools are also useful to be anti-galling even for use in these forging steps. In addition, the tribofilms and carbon agglomerates in Figure 8a can be easily removed from the SiC coating punch surface by cleaning with the use of HCl solvents, for the repetitive use of SiC punch.

## 5. Conclusions

SiC-coated SiC die technology is an anti-galling solution for dry cold forging of titanium work with a high reduction in thickness. No metallic titanium transfer was detected even after continuous forging up to the reduction in thickness by 70%. In situ formed tribofilm on the contact interface of the SiC coating on the titanium work is responsible for this anti-galling forging. As analyzed by SEM–EDX and Raman spectroscopy, the titanium oxide film is in situ formed on the SiC coating in the radial direction from the center of initial contact interface with the flattening of forged titanium work. This film consists of the Magneli-phase intermediate titanium oxides TiO_x_ (1 < x < 2) as well as TiO_2_; this TiOx film works as a lubricious thin layer to reduce the friction by its elasto-plastic flow. In addition, the unbound carbon agglomerates are also formed only at the center of interface which experiences high stress transients during forging. Owing to this in situ formed solid lubricant, the SiC coating surface is prevented from galling by the titanium fresh surface even under high stress states as in forging. This in situ solid lubrication has a close relationship to the carbon supersaturation in the CVD-coated SiC microstructure. Further precise analyses are necessary to describe the effect of stress transients on the carbon isolation from the SiC coating.

In the present study, the sintered SiC block was utilized as a substrate for the SiC CVD coating. Other substrate materials than monolithic SiC are available as a die system for cold and hot forging processes in each application. The graphitic carbon and carbides are candidate materials for CVD SiC coating to yield an anti-galling die system. The warm or hot forging behavior when the holding temperature is less than 673 K, is nearly the same as this cold forging process. Toward the high temperature forging, the die substrate material is appropriately selected to be free from severe oxidation.
